# Selective inhibition of DNA ligase IV provides additional efficacy to the treatment of anaplastic thyroid cancer

**DOI:** 10.3389/fonc.2024.1323313

**Published:** 2024-02-06

**Authors:** Sathya Neelature Sriramareddy, Majeed Jamakhani, Léa Vilanova, Hélène Brossel, Bernard Staumont, Malik Hamaidia

**Affiliations:** ^1^ Molecular and Cellular Epigenetics, Interdisciplinary Cluster for Applied Genoproteomics (GIGA), University of Liège, Liège, Belgium; ^2^ Molecular Biology (TERRA), University of Liege, Gembloux, Belgium

**Keywords:** anaplastic thyroid cancer, DNA ligase IV, homology directed repair, non-homologous end joining, radiotherapy, chemotherapy, single-cell RNA-seq

## Abstract

**Background:**

Although the incidence of anaplastic thyroid carcinoma (ATC) is low (2.5% of thyroid cancer cases), this cancer has a very poor prognosis (survival rates < 5 months) and accounts for 14–39% of deaths. Conventional therapies based on surgery in combination with radiotherapy or chemotherapy showed limited effectiveness primarily due to the robust and protective DNA damage response in thyroid cancer cells.

**Methods:**

We used single-cell transcriptomic data from patients with different subtypes of thyroid cancer to study expression of genes involved in homologous recombination (HR) and non-homologous end joining (NHEJ) pathways. Then, we investigated the mechanisms of DNA damage and repair in anaplastic (C643 and Hth74) and papillary (TPC-1) thyroid cancer cell lines. The effect of caffeine (inhibitor of ATM and ATR) and UCN-01 (CHK1 inhibitor) was evaluated in cell cycle progression of thyroid cancer cells after γ‐radiation or doxorubicin treatment. The DNA damage response was monitored after staining of phosphorylated γ-H2AX and 53BP1. Reporter plasmids were used to determine the efficacy of double-strand DNA breaks (DSBs) repair by HR and NHEJ in thyroid cancer cells. We evaluated the combination of selective inhibition of the DNA ligase IV by SCR7 and doxorubicin on cellular apoptosis and tumor growth in xenograft murine models of anaplastic thyroid cancer.

**Results:**

Single-cell RNA-Seq showed that NHEJ- and HR-related genes are expressed in ATC and PTC patients. We showed that ATC cells undergo mitosis in the presence of unrepaired DNA damage caused by γ‐radiation and doxorubicin treatment. To proliferate and survive, these cells efficiently repair DNA lesions using homologous recombination (HR) and non-homologous end joining (NHEJ). The combination of SCR7 with doxorubicin, significantly increased apoptosis and impaired ATC tumor growth in a xenograft mouse model compared to doxorubicin monotherapy.

**Conclusion:**

This study shows the therapeutic value of the combination of a DNA ligase IV inhibitor and DNA-damaging agents (doxorubicin and/or γ-radiation) for the treatment of anaplastic thyroid cancer.

## Introduction

Accounting for approximately 1% of newly diagnosed cancer cases, the incidence of thyroid cancer has increased over the past 3 decades by >5% per year (1–4). Thyroid cancers originate either from the follicular epithelium or from neuroendocrine C cells (i.e., follicular and medullary thyroid cancer, respectively). Follicular thyroid cancer can be subdivided into well-differentiated (WDTC), poorly differentiated (PDTC), and anaplastic (ATC) thyroid cancers (5). WDTC, which accounts for 90% of cases, includes papillary (PTC) and follicular (FTC) thyroid cancers. With long-term survival rates of 75-90%, the prognosis of medullary and WDTC thyroid cancers is relatively good (6–10). However, a significant proportion (10-35%) of patients relapse and lose the ability to uptake radioiodine-131 in tumors (5, 11–14). The main treatment for ATC and relapsing WDTC includes surgery, cytotoxic treatment (e.g., doxorubicin or cisplatin), and external beam radiation therapy (EBRT). Therapies that target cancer cells carrying the BRAFV600E mutation (dabrafenib/trametinib) have been recently approved for ATC but are associated with significant toxicities (15, 16). Although frequent in PTC, other genomic modifications, such as RET/PTC rearrangement, are uncommon in ATC. With median survival rates varying from 9 weeks to 5 months (5, 11–13, 17, 18), ATC is highly lethal and requires more efficient therapies.

Radiation therapy and topoisomerase inhibitors (e.g., doxorubicin) mainly induce DNA double-strand breaks (DSBs) in tumors. A critical component of radio resistance and chemoresistance is the ability of cancer cells to repair DNA damage. Indeed, DSBs initiate signaling and repair pathways orchestrated by sensors, transducers, and effectors of the DNA damage response (DDR) (19–21). DSBs induced by ionizing radiation and topoisomerase inhibitors can be repaired by homologous recombination (HR) and non-homologous end joining (NHEJ). HR is error-free repair pathway and requires DNA pairing with a homologous chromatid that is only available in late S and G2 phases of the cell cycle. However, NHEJ pathway can be initiated outside S and G2 phase and require a limited sequence homology. The NHEJ is initiated by the Ku70/Ku80 complex, which interacts with DSBs and recruits other components of the repair pathway, including the DNA-dependent protein kinase catalytic subunit (DNA-PKcs), endonuclease Artemis, DNA ligase IV, X-ray repair cross-complementing protein 4 (XRCC4) and polymerases μ and λ (Pol μ and Pol λ) (22). Upon recruitment to the DSB, DNA-PKcs undergoes autophosphorylation and activates Artemis, which then degrades DNA ends to produce short overhangs (≤4 nucleotides) between the strands that facilitate end joining. Upon activation by XRCC4, DNA ligase IV initiates end joining by transferring AMP to the 5′ end of one of the strands at the DSB. Covalent DNA ligation further requires the removal of AMP by aprataxin. Pol μ polymerizes short regions of microhomology for subsequent base pairing in a template‐independent manner. Pol λ primarily promotes the ligation of terminally compatible overhangs that require fill-in synthesis (22). XRCC4-like factor (XLF) stimulates the ligation of short incompatible 3′ overhangs, while the paralog of XRCC4 and XLF (PAXX) promotes the joining of blunt ends. Therefore, NHEJ is the predominant mechanism to process most ionizing radiation-induced DSBs in thyroid cancer cells. However, NHEJ is error-prone process that can be responsible of genome instability or chromothripsis in response to DNA damaging therapies. These genomic rearrangements can increase thyroid cancer aggressiveness via the loss of a tumor suppressor gene and/or oncogene amplification (23). Therapeutic approaches that use pharmacological inhibitors targeting tyrosine kinase receptors (TKIs), BRAF V600E, Mitogen-activated kinases, mTOR, anaplastic lymphoma kinase, tropomyosin receptor kinases are proposed to reduce radio- and chemoresistance of thyroid cancer (14). Another strategy to increase tumor sensitivity to DNA damaging agents is to interfere DSBs repair pathways via the use of selective inhibitors of PARP superfamily (e.g. Niraparib, Olaparib), DNA-PKcs (e.g. NU7441, M3814 or Nedisertib, AZD7648, M9831 or VX-984, KU-0060648), CHK1 (e.g. GDC-0575, MK-8776, Prexasertib), ATM (AZD0156, M3541), ATR (Ceralasertib, Berzosertib), WEE1 (Adavosertib) and DNA ligase IV (SCR7, NU7026) (24, 25). In this context, the objective of this study is (i) to evaluate the regulation of cell cycle and DSB repair activity (ii) to explore the therapeutic potential of DNA ligase IV selective inhibition after treatment of anaplastic thyroid cancer cells (C643 and Hth74) with conventional DNA damaging agents used for the treatment of thyroid cancer.

## Methods

### Cell cultures

Thyroid cancer cell lines C643 and Hth74 (anaplastic thyroid cancer) and TPC-1 (papillary thyroid cancer), provided by Karin Forsberg Nilsson (Uppsala University, Sweden), were grown in Dulbecco’s modified Eagle’s medium (DMEM) containing 100 units per ml penicillin, 100 μg/ml streptomycin and 10% fetal bovine serum (FBS). Cells were maintained at 37°C in a humidified incubator containing a 5% CO_2_ atmosphere.

### Cell cycle analysis

Thyroid cancer cells were seeded in 6-well plates (200,000 cells per well). Twenty-four hours later, the cells were γ-irradiated and/or incubated with checkpoint inhibitors (Sigma–Aldrich): UCN‐01 (7-hydroxystaurosporin) resuspended in DMSO and caffeine (1,3,7-trimethylxanthine) dissolved in DMEM by heating (80°C) for 2 hours. After 24 hours, the cells were trypsinized and fixed in 300 µL of PBS-10% FBS and 700 µL of chilled ethanol. Following fixation overnight at -20°C, cells were incubated with RNase A solution (50 µg/mL in PBS with 0.1% Tween 20 (Sigma–Aldrich) for 30 minutes at 37°C. After suspension in propidium iodide (PI, 20 µg/L, Sigma–Aldrich), fluorescence was analyzed with a FACSCalibur flow cytometer (BD Biosciences) using BD CellQuest Pro software.

### Mitotic trap assay

Three hours after γ-irradiation and/or incubation with UCN-01 or caffeine, thyroid cells were treated with 50 nM of Taxol (Bristol-Myers Squibb). After 16 hours of culture, cells were trypsinized, resuspended in PBS containing 10% FBS, and fixed overnight in 70% ethanol at -20°C. After removing ethanol, cells were labeled for 2 hours with an antibody specific for histone H3 phospho‐Ser10 (Cell Signaling Technology, #9701, 1/200) and an anti-mouse immunoglobulin Alexa-488-conjugate (Invitrogen, 1/1000). After RNase A digestion and PI labeling, fluorescence was analyzed with a FACScanto II flow cytometer (BD Biosciences).

### Confocal microscopy

Cells seeded on coverslips were fixed with 4% paraformaldehyde, permeabilized with 0.1% Triton X-100 for 10 minutes, and incubated with 5% bovine serum albumin (BSA). Cells were then labeled for 2 hours with primary antibodies specific for phospho-H2AX (Cell Signaling Technology, #2577, 1/400) or 53BP1 (Abcam, AB172580, 1/200). After nuclear staining with DAPI (Sigma), the cells were visualized with a Leica SP5 confocal microscope. Simultaneously, a similar experiment was conducted in parallel to analyze the mean fluorescence intensity of phospho-H2AX by FACScanto II flow cytometry.

### Immunoblotting

At different times (2, 5, or 24 hours) after 10 Gy γ-irradiation, cells were lysed on ice with RIPA buffer (150 mM sodium chloride, 1% NP-40, 0.5% sodium deoxycholate, 0.1% SDS, 50 mM Tris pH 8.0) containing protease and phosphatase inhibitors: Halt Protease Inhibitor Cocktail (Thermo Fisher Scientific) and 1 mM phenylmethanesulfonyl fluoride (PMSF) (Sigma). After SDS‐polyacrylamide gel electrophoresis (SDS–PAGE), proteins were transferred onto a nitrocellulose membrane and blocked for 1 hour with 5% BSA (Sigma–Aldrich) in TBS (Tris Buffered Saline) supplemented with 0.1% Tween 20. Proteins were labeled overnight at 4°C with antibodies directed against γ-H2AX (Cell Signaling Technology, #2577, 1/1000) or tubulin (Sigma, SAB4500087, 1/1000). After washing with TBS-Tween (0.1%), membranes were incubated with horseradish peroxidase (HRP)-conjugated secondary anti-rabbit antibody (Cell Signaling Technology, #7074, 1/1000) for 1 hour at room temperature. Luminescence was revealed with HRP substrate (Pierce ECL Western Blotting Substrate, Thermo Scientific) using a CCD camera (ImageQuant LAS4000 mini, GE Healthcare Life Sciences) and analyzed with ImageJ software.

### Quantification of DNA repair efficiency

The quantification of DSB repair by HR and NHEJ was based on plasmid reporters provided by Vera Gorbunova (University of Rochester, USA)^37^. The GFP-Pem1 vector contains a GFP open reading frame interrupted by a 3 Kb intron from the Pem1 gene. In the NHEJ sensor, the Pem1 intron contains an additional adenoviral exon that is flanked by inverted HindIII/I-SceI restriction sites. Endonuclease cleavage leads to nonpalindromic incompatible DNA ends that are repaired by NHEJ. Cleavage of the adenoviral exon, transfection into cells, and repair by NHEJ restore GFP expression. In the HR reporter, the first exon of GFP-Pem1 has a 22 bp deletion flanked by I-SceI/HindIII/inverted I-SceI restriction sites. Due to the deletion, NHEJ repair of the restricted plasmid does not restore GFP expression. The HR reporter contains a second copy of the GFP first exon lacking an ATG. HR between the deleted and ATG-mutated copy of GFP by gene conversion restores green fluorescence.

The HR and NHEJ reporter plasmids were digested overnight with I-SceI (VWR) and purified by gel electrophoresis using the QIAGEN gel extraction kit. Linearized plasmid (2 μg) was transfected with Lipofectamine (Thermo Fisher Scientific) into thyroid cancer cell lines together with 0.5 μg of pHcRed (Clontech). After 48 hours of culture under DNA-damaging conditions, cells were analyzed by flow cytometry. The efficiencies of HR and NHEJ pathways were calculated as ratios of GFP and HcRed fluorescence.

### Analysis of apoptosis

TPC-1, C643, and Hth74 cells were cultivated for 48 hours in 6-well plates (10^5^/well) in the presence of SCR7 (Xcess Bioscience), RI-1 (Axon MedChem), and/or doxorubicin (Sigma Aldrich, 200 nM). Cells were harvested, washed in cold PBS, suspended in 100 μL of binding buffer (PE Annexin V Apoptosis Detection Kit, BD Pharmingen), and labeled with 5 μL of Annexin-V FITC + 7-AAD for 15 minutes in the dark. For the analysis of genomic DNA fragmentation, 10^5^ cells were washed twice in 10% FBS-PBS, resuspended in 300 μL of 10% FBS-PBS, and fixed with 700 μL chilled ethanol (100%) at -20°C. After overnight fixation, cells were recovered by centrifugation, washed twice, treated with RNase A (20 μg/ml) for 30 min, and stained for 10 min with PI (50 μg/ml). Fluorescence was analyzed with 585/42 filters in a FACSCalibur (Becton Dickinson).

### Mouse models

Animal experimentation was approved by the Ethical Committee for the use of laboratory animals at the University of Liège (case number 14-1736) and performed according to the Federation of Laboratory Animal Science Association (FELASA) guidelines. The triple transgenic NOD. Cg-Prkdc^scid^ Il2rg^tm1Wjl^ Tg (CMV-IL3, CSF2, KITLG)1Eav/MloySzJ mice, also called NSG-SGM3 mice (provided by animal facility LA2610359), were inoculated subcutaneously into the right and left flanks with 2.10^6^ C643 or Hth74 cells. A total of 200 µL medium suspension containing 50% v/v Matrigel (Basement Membrane Matrix, Corning) was injected in each flank using a 27G needle. Once the average tumor volume reached 50 mm³, mice were randomized into 6 groups (n=5) to minimize weight and tumor size differences. Mice were mock-treated (vehicle) or injected intraperitoneally with doxorubicin (twice per week at 0.5 mg/kg) and/or SCR7 (10 mg/kg twice a week). Tumors were measured biweekly with a digital caliper, and tumor volume was estimated by using the formula (π x length x width^2^)/6.

### scRNA-Seq data processing

The publicly available scRNA-Seq data from 10 ATC tumors, 7 PTC tumors and 6 adjacent normal thyroid tissues with the GEO accession number GSE193581 were used for our study (26). Raw data composed of approximately 71,831 cells were filtered by using quality metrics (percentage of mitochondrial genes) with Scanpy (version 1.9.3). Single cells that had fewer than 200 genes or more than 6,000 genes detected were removed. Doublets were removed from each sample (Scrublet, version=0.2.3). Approximately 40,070 cells passed the quality control. The count matrix was log normalized and Z transformed (scanpy, version 1.9.3). The batch effect was evaluated and corrected (scanorama, version 1.7.3). The scanorama-corrected data were clustered by using the Elbow method, and 30 principal components were retained to determine the number of clusters (k) by using the k-means clustering algorithm. The k-mean value of 10 was used for our study. Clusters were automatically annotated for different cell types (CellTypist, version 1.5.2). Epithelial cells from k-mean clusters were extracted to perform differential gene expression between ATC, PTC and adjacent normal thyroid tissues (normal cells) within the epithelial cluster. Differential gene expression was performed by using the Wilcoxon rank sum test (scanpy, version 1.9.3) of 3 groups of ATC, PTC and normal cells.

### Statistical analysis

Statistical relevance was determined using GraphPad Prism 8. The Shapiro–Wilk test was used to determine the normality of distribution, and the F test was used to determine the equality of variances. Means within a dataset with equal variance were compared by 1-way ANOVA followed by Tukey’s multiple-comparisons test. Statistical significance between non-Gaussian paired distributions was calculated using the nonparametric Friedman’s test followed by Dunn’s multiple-comparisons test. The analysis of tumor growth was performed using 2-way ANOVA and Bonferroni’s post-tests. Survival curves were compared by using a log-rank test (χ2). For Western blot and imaging data, statistical analysis was performed using the nonparametric Friedman’s test followed by Dunn’s multiple-comparisons test. Data were considered statistically significant (*), very statistically significant (**), and highly statistically significant (***) at P < 0.05, P < 0.01, and P < 0.001, respectively.

## Results

### NHEJ- and HR-related genes are differentially expressed in ATC and PTC tumors

**Figure 1:**
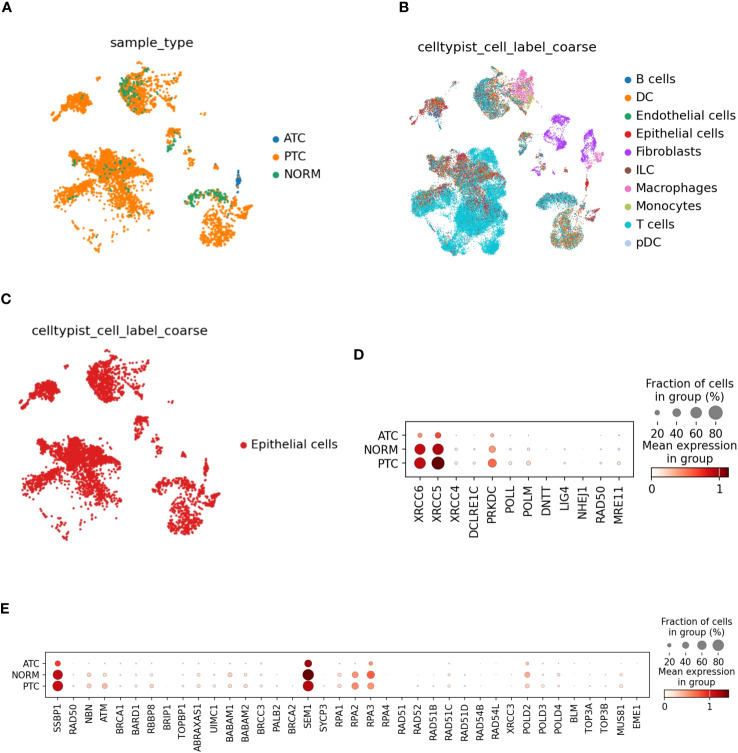
NHEJ- and HR-related genes are differentially expressed in ATC and PTC tumors. **(A)** The publicly available scRNA-Seq data from 10 ATC tumors, 7 PTC tumors, and 6 adjacent normal thyroid tissues with the GEO accession number GSE193581 were used for our study (26). Raw data composed of approximately 71,831 cells were filtered by using quality metrics, and approximately 40,070 cells passed the quality control. The scanorama-corrected data were clustered using the Elbow method, and 30 principal components were retained to determine the number of clusters (k) by using the k-means clustering algorithm (k-mean value 10). The k-mean value of 10 was used for our study. **(B)** Clusters were automatically annotated for different cell types by using CellTypist (version 1.5.2). **(C)** Clustering of cells expressing epithelial markers. **(D, E)** Plot showing mean expression in group and fraction of expressing cells within the epithelial cells cluster of genes involved in HR and NHEJ pathways. The differential gene expression was performed using scanpy (version 1.9.3) and the Wilcoxon rank sum test for 3 groups: ATC, PTC, and normal cells.

We used a publicly available dataset of single-cell transcriptomes from 10 ATC tumors, 7 PTC tumors, and 6 adjacent normal thyroid tissues (GSE193581) to determine the expression of genes involved in NHEJ and HR pathways. A total of 40 070 cells out of 71 831 passed the quality control and were investigated for further analyses. We performed k-mean clustering (k-mean value 10) and automated identification of 10 major cell types found in the tumor microenvironment (**Figures 1A, B**). The differential gene expression was investigated from the epithelial cluster because both normal thyroid cells and tumor cells are of epithelial origin (**Figure 1C**, **Supplementary Table**). We determined the differential expression of genes highly involved in NHEJ and HR pathways (KEGG references: K10980 and map03440, respectively) in tumors (PTC and ATC) and normal thyroid tissues (NORM). NHEJ- (*XRCC6, XRCC5, PRKDC*) and HR‐ (*SSBP1, SEM1, RPA2, RPA3*)-related genes were found to be expressed in both normal follicular and thyroid cancer cells. The mean expression of NHEJ-related genes (*XRCC6, XRCC5, PRKDC*) decreased in ATC epithelial cells compared to normal thyroid and PTC cells. The fraction of cells expressing HR-related genes (*SSBP1, SEM1, RPA2, RPA3*) was reduced but with a similar mean expression compared to normal cells and PTC (**Figures 1D, E**). These data suggest that both the HR and NHEJ pathways exist in ATC and cooperate and/or compete for DSB repair.

These results show that HR- and NHEJ-related genes are expressed by PTC and ATC cells from patients.

### Cell cycle checkpoint inhibitors abrogate G2/M arrest in γ−irradiated thyroid cancer cells

To determine optimal experimental conditions, the cytotoxicity of checkpoint kinase inhibitors (caffeine, UCN-01) and γ-radiation was evaluated in 3 cell lines pertaining to different histological subtypes of thyroid cancer: C643, Hth74 (anaplastic) and TPC-1 (papillary) cells (27–29). Cells were cultivated for 24 hours in the presence of increasing concentrations of caffeine (an inhibitor of ATM and ATR: 0.5 mM-5 mM) or UCN-01 (an inhibitor of CHK1: 0-100 nM) and/or after γ‐radiation (0-10 Gy). Based on the dose–response relationship (**Supplementary Figure 1**), subtoxic concentrations were selected for further analyses (i.e., 50 nM for UCN-01 and 2.5 mM for caffeine).

**Figure 2:**
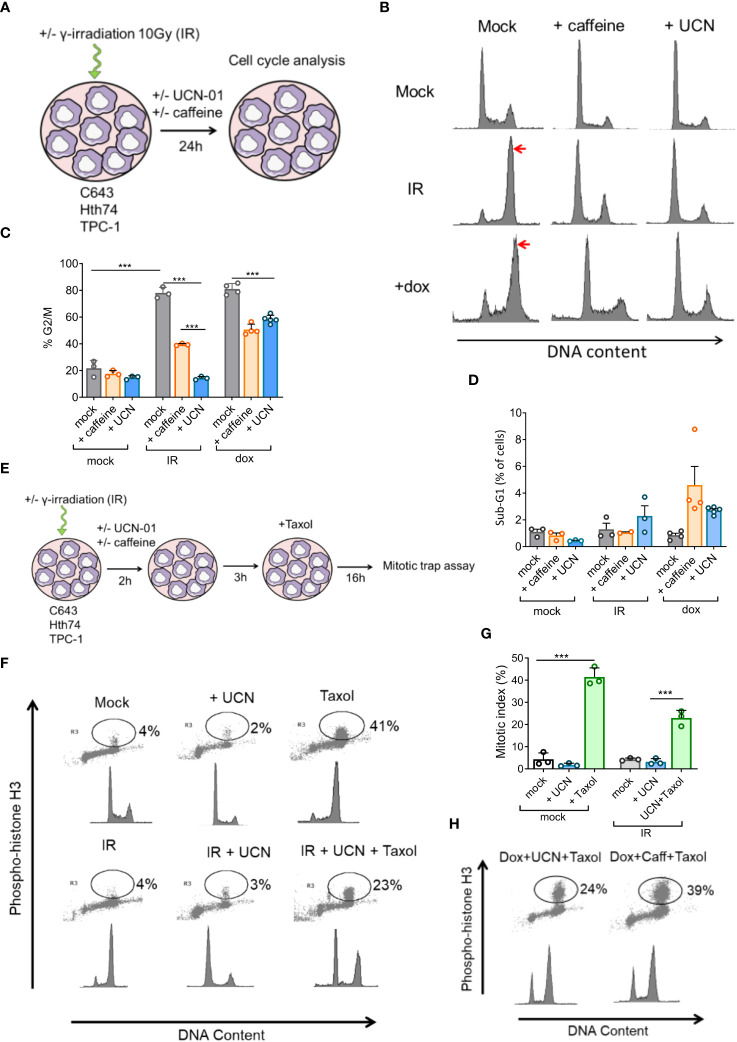
Thyroid cancer C643 cells bypass the mitotic checkpoint induced by γ−irradiation in the presence of UCN-01 or caffeine. **(A)** Thyroid carcinoma cells (C643) were γ-irradiated with 10 Gy and/or cultivated with caffeine (2.5 mM) or UCN-01 (50 nM). After 24 hours, the cells were fixed and permeabilized with ethanol and labeled with propidium iodide. **(B)** The cell cycle profiles were analyzed by flow cytometry and histograms indicating the fluorescent profile of propidium iodide (PI) corresponding to cell DNA content (x-axis) and the cell count (y-axis). **(C, D)** Histogram showing percentages of cells in G2/M and sub-G1 calculated from 4 independent experiments. **(E)** Mitotic trap assay. Three hours after γ-irradiation (IR), UCN-01 (50 nM, UCN-01), and/or caffeine (2.5 mM, Caff), C643 thyroid cells were incubated with taxol at 50 nM. Sixteen hours later, the cells were fixed and permeabilized with ethanol. Cells were stained with a histone 3 anti-phosphoserine 10 (H3pSer10) antibody and PI and analyzed by flow cytometry. **(F)** Histogram and dot plot indicating on the x-axis fluorescence associated with PI (DNA content) and on the y-axis the fluorescence associated with staining of anti-H3pSer10. **(G)** Histogram indicating the percentage of H3pSer10-positive cells from three independent experiments. **(H)** C643 cells were analyzed for the staining of H3pSer10 and PI as described in panel A except that doxorubicin (500 nm) was used instead of γ-irradiation. Each bar represents the mean +/- SD. Statistical significance was determined by one-way analysis of variance (ANOVA) followed by Tukey’s multiple comparisons test. Data were considered statistically significant (*), very statistically significant (**), and highly statistically significant (***) at P < 0.05, P < 0.01, and P < 0.001, respectively.

To investigate checkpoint control, the 3 cell lines were exposed to 10 Gy of γ-irradiation (IR) and analyzed by flow cytometry (**Figure 2A**). After 24 hours, C643 cells accumulated in G2/M (arrows on **Figure 2B**), indicating that checkpoint control prevented entry into mitosis. DNA damage resulting from γ-irradiation blocked mitosis in C643 (78%), TPC-1 (43%), and Hth74 (44%) cells (**Figure 2C**, **Supplementary Figures 2, 3**). In the presence of caffeine, the percentages of γ-irradiated cells in G2/M were reduced to 39%, 21%, and 32% in C643, TPC-1, and Hth74 cells, respectively (**Figure 2C**, **Supplementary Figures 2, 3**). A similar effect was obtained with another checkpoint inhibitor (UCN-01), although less efficiently in TPC‐1 cells. Under these conditions, apoptosis evaluated by DNA fragmentation (sub-G1 peak) and polyploidy (>G2/M) remained negligible (**Figure 2D**, **Supplementary Figures 2–4**).

These data thus suggest that checkpoint inhibitors allow γ-irradiated thyroid cells to escape G2/M arrest and enter the G1 phase.

### γ−Irradiated thyroid cells undergo mitosis in the presence of caffeine and UCN-01

To confirm that γ-irradiated cells underwent effective mitosis in the presence of checkpoint inhibitors, C643 cells were labeled for histone 3 phosphoserine 10 (H3pSer10) and analyzed for their DNA content by flow cytometry (**Figure 2E**). γ-Irradiation increased the percentages of G2/M cells but did not significantly modify H3pSer10 labeling (4%). In contrast, the spindle inhibitor Taxol increased the percentages of H3pSer10-positive cells from 4% (control) to 41% (**Figures 2F, G**). Since Taxol inhibits the cytokinesis of mitotic cells, it is estimated that a significant proportion of C643 cells re-entered G1 after 24 hours. In the presence of UCN-01 and Taxol, 23% of γ-irradiated C643 cells were positive for H3pSer10. Checkpoint abrogation by UCN-01 thus allowed a significant proportion of γ-irradiated cells to undergo mitosis (20%, the difference between 23% and 3%) (**Figures 2F, G**). Similar conclusions were drawn using doxorubicin, a chemotherapeutic compound used in patients with advanced thyroid cancer (**Figure 2H**). It thus appears that when cell cycle checkpoints are inhibited by UCN-01, C643 cells divide during the 24-hour period despite being γ-irradiated. These conclusions were extended to another cell line (Hth74) treated with caffeine (**Supplementary Figure 5**).

### γ−Irradiation is genotoxic to anaplastic thyroid cancer cells

**Figure 3:**
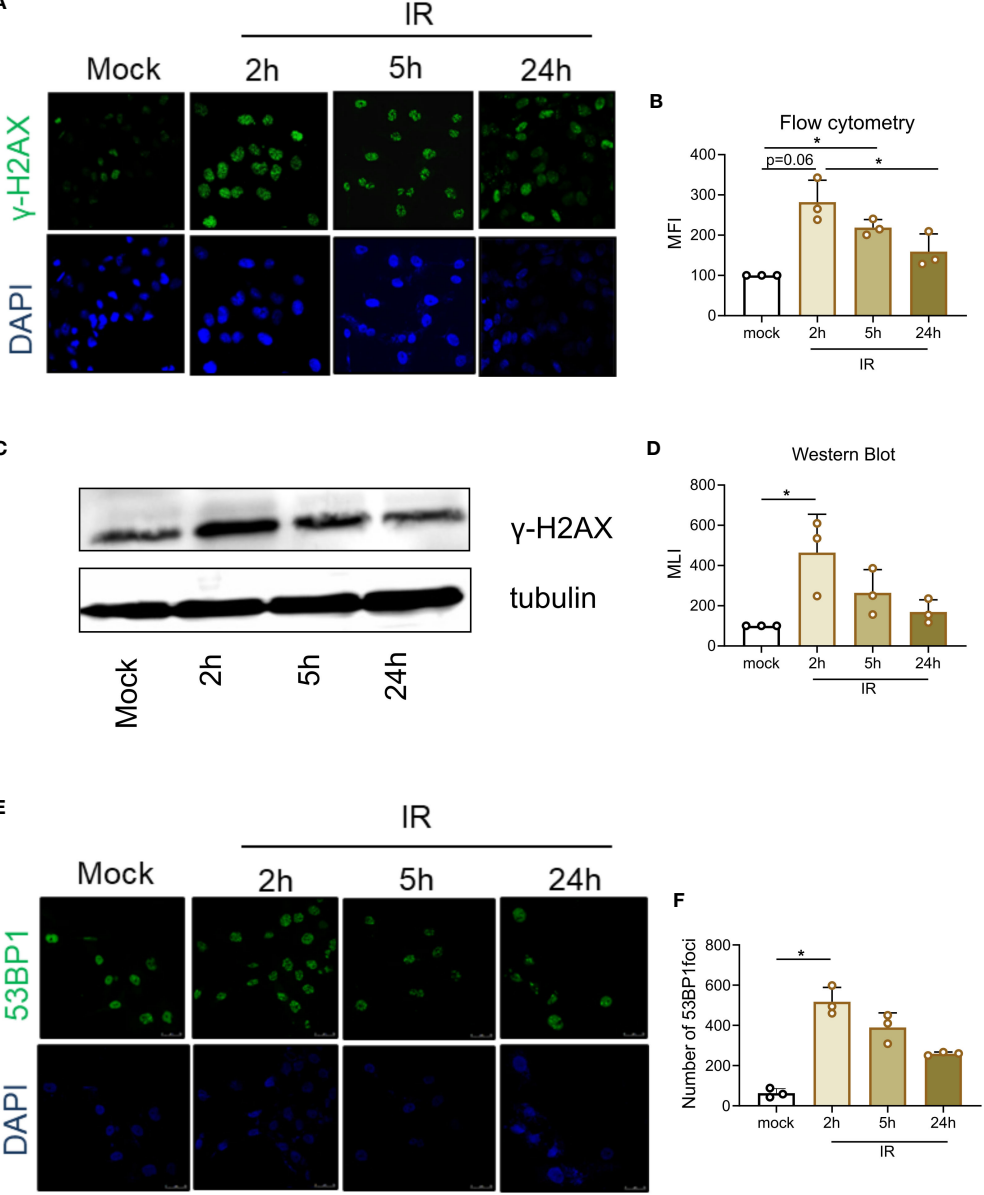
γ−Irradiation is genotoxic to thyroid cancer cells. **(A)** At 0, 2, 5, and 24 hours post-irradiation, C643 cells were analyzed by confocal microscopy after labeling with an anti-γ-H2AX antibody and an Alexa Fluor 488 conjugate (green fluorescence). Nuclei were stained with DAPI (blue fluorescence). **(B)** C643 cells were treated as described in panel A and analyzed by flow cytometry. The mean fluorescence intensities (MFI) of γ-H2AX foci were normalized to the control arbitrarily set to 100. Data represent the means of three independent experiments. Statistical significance was determined by using the nonparametric Friedman test followed by Dunn’s multiple comparisons. **(C)** Immunoblot analysis of γ-irradiated C643 cells labeled with antibodies directed against γ-H2AX and tubulin. **(D)** Quantification of immunoblot luminescence intensities normalized to mock calculated from 3 independent experiments. Statistical significance was determined by using the nonparametric Friedman test followed by Dunn’s multiple comparisons. **(E)** Cells were γ-irradiated and analyzed by confocal microscopy after labeling with an anti-53BP1 antibody and an Alexa Fluor 488 conjugate (green fluorescence). Nuclei were stained with DAPI (blue fluorescence). **(F)** 53BP1 foci were quantified at 0-24 hours post-irradiation from 3 independent experiments. Each bar represents the mean +/- SD. Statistical significance was determined by using the nonparametric Friedman test followed by Dunn’s multiple comparisons. The data represent the means of three independent experiments. Data were considered statistically significant (*) at P < 0.05.

Data from **Figure 2** show that a significant proportion of γ-irradiated cells survive and undergo mitosis in the presence of checkpoint inhibitors. To evaluate the extent of DNA damage, cells were stained for Ser139 phosphorylation of H2AX (γ-H2AX). As shown in panel A of **Figure 3**, γ-irradiation induced a rapid increase of γ-H2AX foci in C643 cells, indicating the onset of DNA damage. The maximum number of *γ*-H2AX foci enumerated at 2 hours gradually decreased at 5 and 24 hours post-irradiation. Similar observations were confirmed by measuring the mean intensity of fluorescence (**Figure 3B**) and by immunoblotting (**Figures 3C, D**). Finally, another marker of DNA damage repair, 53BP1, further validated the conclusions (**Figures 3E, F**).

Taken together, these results show that, as expected, a γ-irradiation dose of 10 Gy induces significant damage, suggesting that thyroid cancer cells can efficiently repair their DNA in response to *γ*-irradiation.

### DNA double-strand breaks are efficiently repaired in thyroid cancer cells

**Figure 4:**
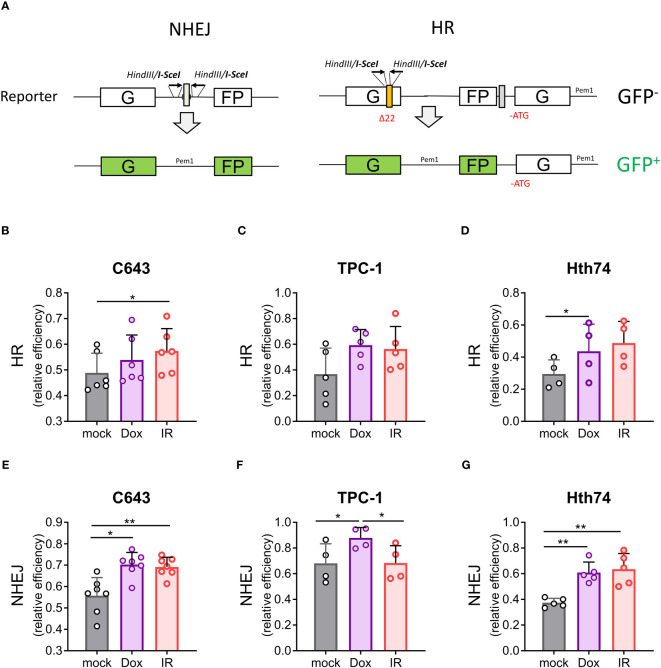
ATC and PTC cells efficiently repair DNA double-strand breaks caused by γ-irradiation and doxorubicin. **(A)** Reporter constructs for the analysis of DNA DSB repair by NHEJ and HR as described in (30). C643, Hth74, and TPC-1 cells were transfected with HR or NHEJ reporter plasmids. DNA double-strand breaks were induced by γ-irradiation or treatment with doxorubicin (300 nM). **(B–G)** Quantification of HR and NHEJ repair efficiencies was based on GFP reporter plasmids and calculated as described in the Materials and Methods. The data represent quantification from n>4 independent experiments. Each bar represents the mean +/- SD. Statistical significance was determined by one-way analysis of variance (ANOVA) followed by Tukey’s multiple comparisons test. Data were considered statistically significant (*), and very statistically significant (**), at P < 0.05, and P < 0.01 respectively.

The efficiency of these 2 repair pathways was quantified using GFP-based reporter vectors in C643, TPC-1, and Hth74 cells. In these systems, functional GFP is expressed when a DSB (created *in vitro* by the I-SceI endonuclease) is repaired in cellulo by HR or NHEJ (**Figure 4A**). Flow cytometry data were normalized to an internal control (pHcRed) to eliminate variations in transfection efficiencies. Absolute rates of DNA repair efficiency were calculated based on the ratio between the number of GFP^+^ cells generated by the HR and NHEJ reporters and the number of HcRed^+^ cells. Relative repair efficiencies were obtained by normalizing these ratios with a control GFP plasmid. As predicted, C643, TPC-1, and Hth74 cells efficiently repaired the DNA lesions induced by *γ*-irradiation using both HR and NHEJ pathways (**Figures 4B–G**). Repair efficiencies significantly increased in most experimental settings after *γ*-irradiation except for NHEJ in TPC-1 cells and HR in Hth74 and TPC-1 cells. Similar conclusions were obtained with another DNA-damaging agent (doxorubicin), except for HR in C643 and TPC-1 cells.

These results show that thyroid cells are able to actively repair genomic lesions by HR and NHEJ.

### Inhibition of double-strand break repair induces apoptosis of thyroid carcinoma cells

**Figure 5:**
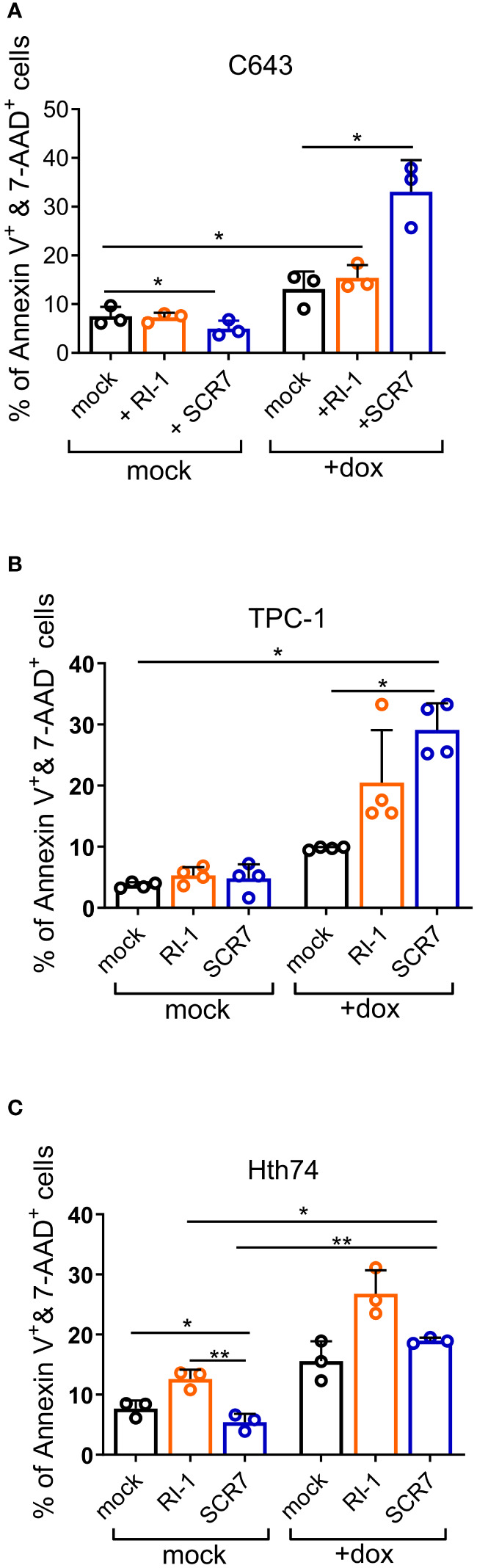
Inhibition of double-strand break repair induces apoptosis of thyroid carcinoma cells. **(A)** C643, **(B)** TPC-1 and **(C)** Hth74 74 cells were treated with LIG4 inhibitor (SCR7; 200 µM) or RAD51 inhibitor (RI-1; 100 µM) and/or doxorubicin (200 nM, Dox). After 48 hours, apoptotic cells were labeled with Annexin V FITC and 7AAD and analyzed by flow cytometry. The data represent the mean from 3 independent experiments. Each bar represents the mean +/- SD. Statistical significance was determined by one-way analysis of variance (ANOVA) followed by Tukey’s multiple comparisons test. Data were considered statistically significant (*), and very statistically significant (**) at P < 0.05, and P < 0.01, respectively.

Since thyroid cancer cells require efficient DNA repair, we evaluated the effect of NHEJ and HR inhibitors in the presence of agents inducing DSBs, such as doxorubicin. RI-1 is a small molecule that inhibits the central recombination protein RAD51 involved in the gene conversion pathway of HR (RI-1) (31). SCR7 interferes with the binding of DNA ligase IV (LIG4) to DNA and thereby inhibits NHEJ (32). The pro-apoptotic effect of these 2 inhibitors on thyroid cell lines was evaluated by Annexin V and 7-AAD labeling. Each inhibitor used alone had only a minor effect on the survival of C643, TPC-1, and Hth74 cells (**Figures 5A–C**). Similarly, RI-1 was inefficient in significantly increasing doxorubicin-induced apoptosis in C643 and TPC-1 cells. In contrast, SCR7 combined with doxorubicin efficiently promoted apoptosis of C643 and TPC-1 cells (**Figures 5A, B**). However, the combination of doxorubicin with selective inhibitors did not significantly affect the apoptosis of Hth74 cells (**Figure 5C**). The onset of apoptosis evaluated by Annexin-V and 7-AAD labeling was confirmed by measuring DNA fragmentation (sub-G1 peak) in different cell lines (**Supplementary Figure 6**).

Taken together, these results demonstrate that apoptosis can be induced in thyroid cells by a combination of a DSB-inducing agent and inhibitors of DNA repair.

### Inhibition of DNA repair impairs tumor growth in mouse models

**Figure 6:**
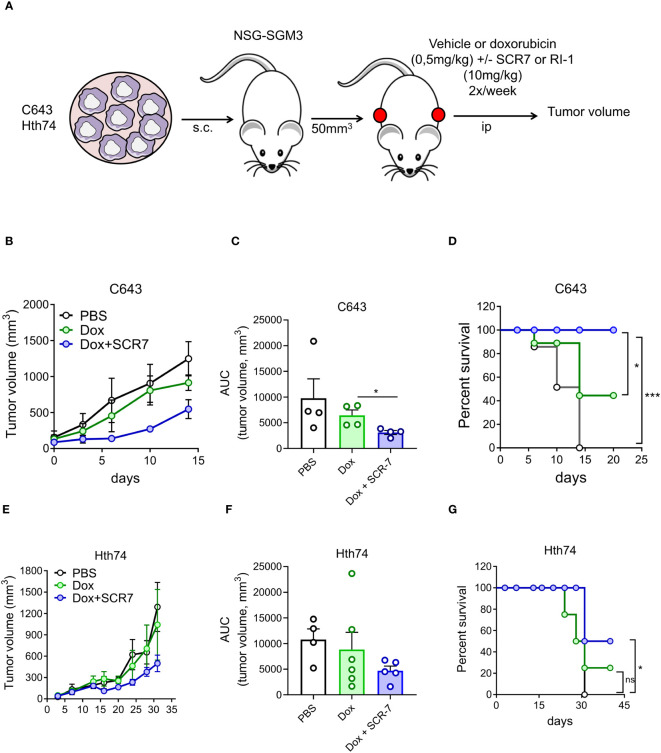
SCR7 combined with doxorubicin inhibits tumor growth in a thyroid carcinoma xenograft mouse model. **(A)** Tumor growth of C643 and Hth74 thyroid carcinoma cells was evaluated after subcutaneous injection in immunocompromised NSG3GS mice in each flank. Mice were treated intraperitoneally with PBS, doxorubicin (0.5 mg/kg twice a week, Dox), or SCR7 (10 mg/kg twice a week). **(B, E)** Graph indicating the mean tumor volume (mm^3^) measured bi-weekly over the time from n=5 mice. **(C, F)** Histogram showing the mean area under individual tumor growth curves (AUCs). Statistical significance was determined by the nonparametric Kruskal–Wallis test followed by Dunn’s multiple comparisons. **(D, G)** represent the corresponding survival curves. Statistical significance was determined by performing a chi-square log-rank (Mantel–Cox) test. Each bar represents the mean +/- SEM. Data were considered statistically significant (*), and highly statistically significant (***) at P < 0.05, and P < 0.001, respectively.

Since the pro-apoptotic activity of SCR7 in combination with doxorubicin depends on the cell type, its therapeutic potential was evaluated in 2 mouse models of ATC. Immunodeficient NSG‐SGM3 mice were inoculated subcutaneously with anaplastic C643 and Hth74 thyroid cancer cells. Once the tumor reached a mean volume of 50 mm³, mice were injected twice per week with SCR7 (10 mg/kg) or/and doxorubicin (0.5 mg/kg) (**Figure 6A**). When used as a single agent, doxorubicin showed no antitumor activity compared to the vehicle (**Figures 6B, C, E, F**). In contrast, the combination of SCR7 with doxorubicin reduced tumor growth and prolonged the survival of C643-inoculated mice (**Figures 6B–D**). The combination of SCR7 and doxorubicin caused a minor tumor reduction but significantly increased Hth74-inoculated mice survival compared to doxorubicin alone (**Figures 6E–G**).

We conclude that the inhibition of DNA ligase IV increased the control of tumor growth after doxorubicin treatment in xenograft murine models of ATC.

## Discussion

Thyroid tumors are commonly treated by radiotherapy by using radioactive iodine (RAI) to cause double-strand breaks (DSBs) (20, 21, 33). The elevated number of DSBs destroys the genome integrity of cancer cells and causes their elimination. However, non-homologous end joining (NHEJ) and homologous recombination (HR), 2 representative DSB repair pathways, are effective in maintaining genetic information and favor thyroid cancer resistance to therapies using DNA damaging factors. Radiotherapy and chemotherapy resistance have been shown to lead to cancer relapse and poor prognosis of cancer patients (34, 35). The identification of DSB repair inhibitors is urgently needed to improve the outcomes of these therapies. In principle, inhibition of cell cycle checkpoint kinases is thus predicted to ameliorate radiosensitization (36–39).

In this context, we evaluated the effect of 2 checkpoint inhibitors, caffeine (a methylxanthine alkaloid) and UCN-01 (an indolocarbazole ATP analog), on thyroid cancer cell lines (38–40). We used C643, Hth-74 and TPC-1 cell lines cells that are respectively characterized by *HRAS* mutation, *NF1* mutation and *RET/PTC1* rearrangement (29). *HRAS* and *NF1* mutations are commonly detected in ATC (respectively 10-20% and 9% of ATC tumors) (41, 42). These cells are also found to be mutated for *TP53* gene (29). Previous studies showed that both UCN-01 and caffeine increased the sensitivity of tumor cells to chemotherapy (cisplatin, camptothecin, doxorubicin) and γ-radiation (43–46). Our present study shows that despite successful checkpoint abrogation, γ-radiation in combination with caffeine or UCN-01 has a minor effect on cell survival. We observed that a significant fraction of thyroid cancer cells resumed the cell cycle and survived, thus extending observations in different cancer types (47–49). When thyroid tumor cells are released from G2 arrest by checkpoint inhibitors, cells have the possibility either to repair DNA damage before entering mitosis or to undergo polyploidy. If cells bypass the mitotic checkpoint without repairing DNA lesions, they undergo mitotic catastrophe and cell death due to improper segregation of fragmented chromosomes (50–52). Unexpectedly, we did not observe significant levels of micronuclei or polyploidy in any of the treatment combinations (**Supplementary Figures 2, 3**). In the absence of apoptosis, mitotic catastrophe, or polyploidy, we hypothesized that thyroid cancer cells may bypass mitotic checkpoints and survive. Using a mitotic trap assay, we demonstrated that a significant proportion of cells successfully underwent mitosis in the presence of caffeine or UCN-01 (**Figure 2**, **Supplementary Figure 5**). The kinetics of γ-H2AX and 53BP1 labeling indicated that double-strand DNA lesions induced by irradiation are rapidly resolved (**Figure 4**), consistent with other reports (47–49).

**Figure 7:**
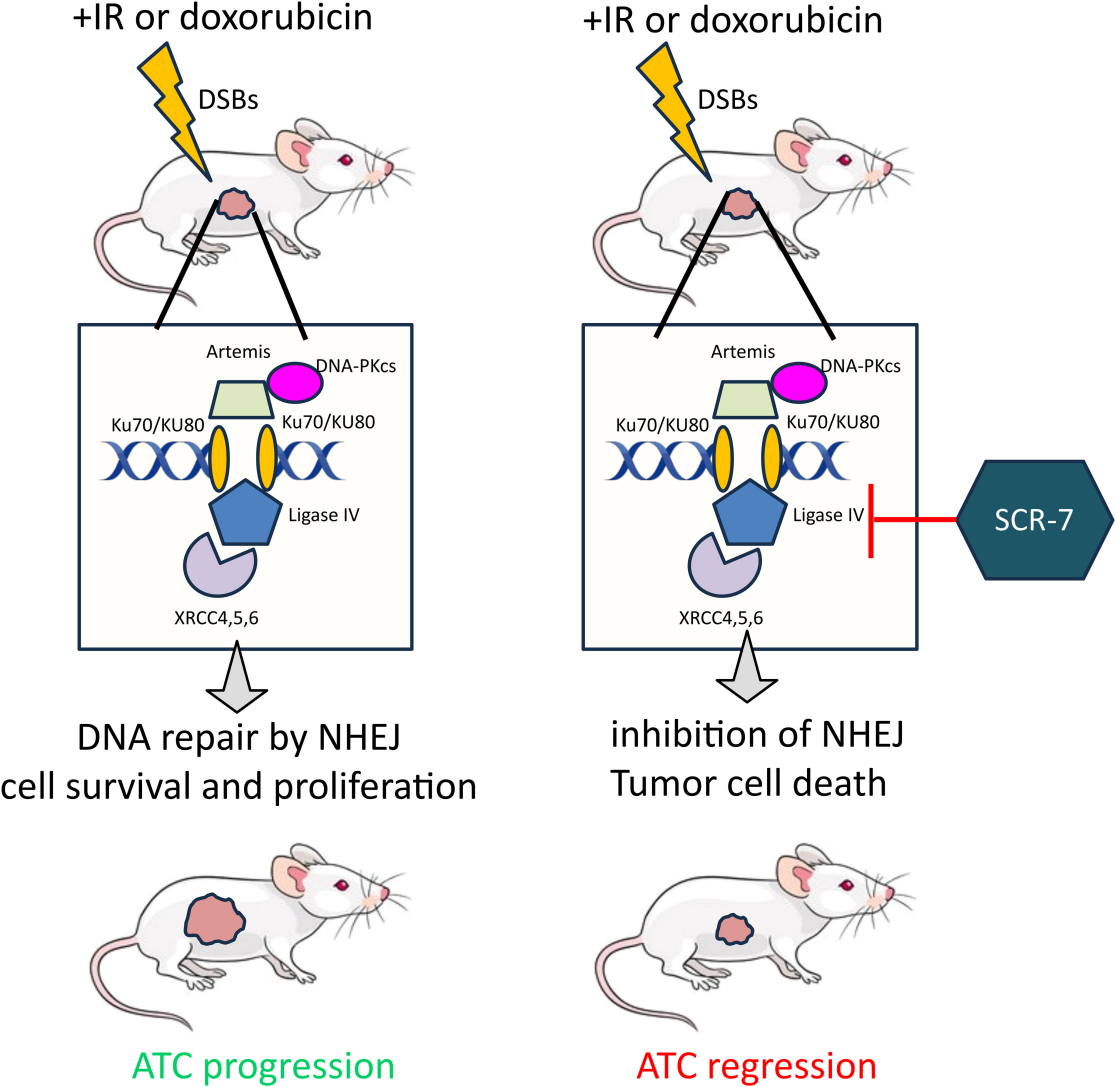
A model showing tumor control after the combination of a selective LIG4 inhibitor (SCR7) and doxorubicin in a mouse xenograft model of anaplastic thyroid cancer. Treatment with doxorubicin causes double-strand DNA breaks (DSBs) which are rapidly repaired by the non-homologous end-joining pathway (NHEJ). NHEJ is initiated by the recruitment of the Ku complex composed of Ku70 and Ku80 to double-strand DNA ends. Once attached, DNA-PKcs is recruited for the protection of DNA ends and the formation of the DNA-PK holoenzyme. DNA-PK activates the XRCC4-LIG4-XLF complex for the final ligation of DNA ends. Once repaired, thyroid cancer cells resume the cell cycle, causing tumor progression. The combination of doxorubicin and selective inhibitor of DNA ligase IV (SCR7) reduced tumor growth by impairing the NHEJ repair pathway of DSBs caused by doxorubicin. The accumulation of DSBs causes cell death and tumor regression.

We used publicly available scRNA-seq data to analyze the expression of key genes involved in the HR and NHEJ pathways (26). NHEJ- (*XRCC6, XRCC5, PRKDC*) and HR- (*SSBP1, SEM1, RPA2, RPA3*) related genes are expressed by both normal follicular and thyroid cancer cells. NHEJ-related genes are more highly expressed in PTC than in ATC. However, the level of HR-related gene expression is similar between PTC and ATC. These data suggest that both HR and NHEJ pathways exist in ATC and cooperate and/or compete for DSB repair in patients. We observed that the DSB repair capacity using HR and NHEJ globally increased after γ-radiation or doxorubicin treatment in ATC and PTC cells. HR requires a template and numerous enzymes and can perform an error-free DDR compared to NHEJ (53). Template-independent NHEJ is predominant and can lead to mutations (error-prone) in genes involved in negative regulation of the cell cycle, thus promoting therapy resistance. NHEJ is initiated by XRCC5 (Ku80) and XRCC6 (Ku70), which directly interact with DSB ends (54). DSB ends are protected by DNA-dependent protein kinase (DNA-PKcs) before ligation by the XRCC4-XLF complex and DNA ligase IV (LIG4) (**Figure 7**) (53). Mutations and/or the loss of factors directly involved in the NHEJ pathway have been associated with increased sensitivity to DNA-damaging agents (55). The use of selective inhibitors of NHEJ repair is a groundbreaking therapeutic approach to promote genome instability by minimizing the dose of chemotherapy and radiation therapy. In fact, overexpression of DNA-PKcs, LIG4 and XRCC4 is correlated with poor prognosis in several cancer types, such as esophageal cancer, colorectal cancer, bladder cancer, ovarian cancer, and hepatocellular cancer (56–60). Targeting DNA-PKcs catalytic activity with potent selective inhibitors (NU7441 and KU-0060648) to increase efficacy of radiotherapy was extensively evaluated in pre-clinical and clinical trials (NCT02516813, NCT03770689, NCT04555577, NCT04533750, and NCT03907969) (61). However, the use of DNA ligase IV inhibitors for cancer sensitization to DNA damaging agents is poorly exploited (53). It has been shown that patients with homozygous mutations in DNA ligase IV are characterized by hyper-radiosensitivity (62–64). scRNA-seq analysis showed reduced expression of the *LIG4* gene at the mRNA level in normal follicular thyroid cells and thyroid cancer cells, but its activity is tightly regulated at the protein level by XRCC4 and DNA-PKcs (65). Furthermore, the DNA ligase IV is detected at protein level in 50% of thyroid cancer patients (https://www.proteinatlas.org/ENSG00000174405-LIG4/pathology) thus making DNA ligase IV an attractive target to develop new antiproliferative agents. The first generation of DNA ligase IV inhibitors (L189, IC50 value 5+/- 2µM) showed low inhibition efficacy and specificity compared to SCR7 inhibitor (32, 66). Recently, it has been shown that SCR7 can also inhibit the DNA ligase IIIα/XRCC1 (67). The combination therapy of SCR7 and chemotherapy was shown to enhance melphalan cytotoxicity in patients with multiple myeloma (68). Doxorubicin when administered with SCR7 showed an increased efficacy in cervical cancer compared to monotreatment (69). In this report, we evaluated the combination therapy SCR7/doxorubicin for the treatment of thyroid cancer. Doxorubicin monotherapy showed effective control of ATC growth in patients when used in the initial stages. However, repeated administration of doxorubicin is commonly associated with cancer drug resistance and adverse effects (e.g. cardiotoxicity, stomatitis, bone marrow aplasia) (70, 71). NSG immunocompromised mice were shown to be sensitive to doxorubicin therapy and resulted in gastrointestinal and hepatic injuries and cardiotoxicity (72). Our preclinical study was conducted by a well-tolerated dose of doxorubicin (0.5 mg/kg, twice a week) which represents 1/8 the dose of studies using xenograft models (72). We showed that the combination of a low dose of doxorubicin with SCR7 significantly increased cell apoptosis and enhanced tumor control in C643 xenograft model. The drug resistance associated with Hth74 cells is not understood and is probably caused by the presence of *NF1* and *TP53* mutations (73). Our data showed an increased sensitivity of TPC-1 cells (*RET/PTC1* rearrangement) to doxorubicin in presence of the DNA ligase IV inhibitor *in vitro*. The therapeutic potential of the DNA ligase IV inhibitor should need further validation on thyroid cancer cell lines characterized by different cancer genetic drivers (e.g. BRAF p.V600, NRAS p.Q61K) (29). A broader panel of cell lines, in particular BRAFV600E positive ATCs (e.g. 8505C, SW1736), would increase the relevance of the study. By increasing DSBs, the combination therapy doxorubicin/SCR7 can be used to increase the tumor mutational burden thereby supporting anti-tumor immunity (74, 75). We showed that the administration of SCR7 and doxorubicin offers a promising strategy for the treatment of papillary and anaplastic thyroid cancer.

## Conclusions

Taken together, this evidence suggests that SCR7 is a promising candidate to reduce thyroid cancer resistance to multimodal therapy. Of note, adequate timing and dosage of combination therapy is required to prevent therapy-related secondary cancers. While new inhibitors are becoming available and are currently evaluated in clinical trials, our results support the proof-of-concept of a strategy interfering with NHEJ repair in advanced thyroid cancer.

## Data availability statement

The datasets presented in this study can be found in online repositories. The names of the repository/repositories and accession number(s) can be found in the article/**Supplementary Material.** We used a publicly available dataset of single-cell transcriptomes (GSE193581): https://www.ncbi.nlm.nih.gov/geo/query/acc.cgi?acc=GSE193581.

## Ethics statement

Ethical approval was not required for the studies on humans in accordance with the local legislation and institutional requirements because only commercially available established cell lines were used. The animal study was approved by Ethical Committee for the use of laboratory animals at the University of Liège. The study was conducted in accordance with the local legislation and institutional requirements.

## Author contributions

SS: Conceptualization, Investigation, Writing – review & editing, Methodology. MJ: Investigation, Methodology, Writing – review & editing. LV: Writing – review & editing. HB: Writing – review & editing. BS: Writing – review & editing. MH: Conceptualization, Funding acquisition, Investigation, Methodology, Supervision, Writing – original draft, Writing – review & editing.
