# Supplementary material for: Selective inhibition of DNA ligase IV provides additional efficacy to the treatment of anaplastic thyroid cancer
Source: Front Oncol. 2024 Feb 6;14:1323313. doi: 10.3389/fonc.2024.1323313 (PMC10876873; doi:10.3389/fonc.2024.1323313)
Supplement: Supplementary file 2 [file Presentation_2.pptx]

## Slide 1
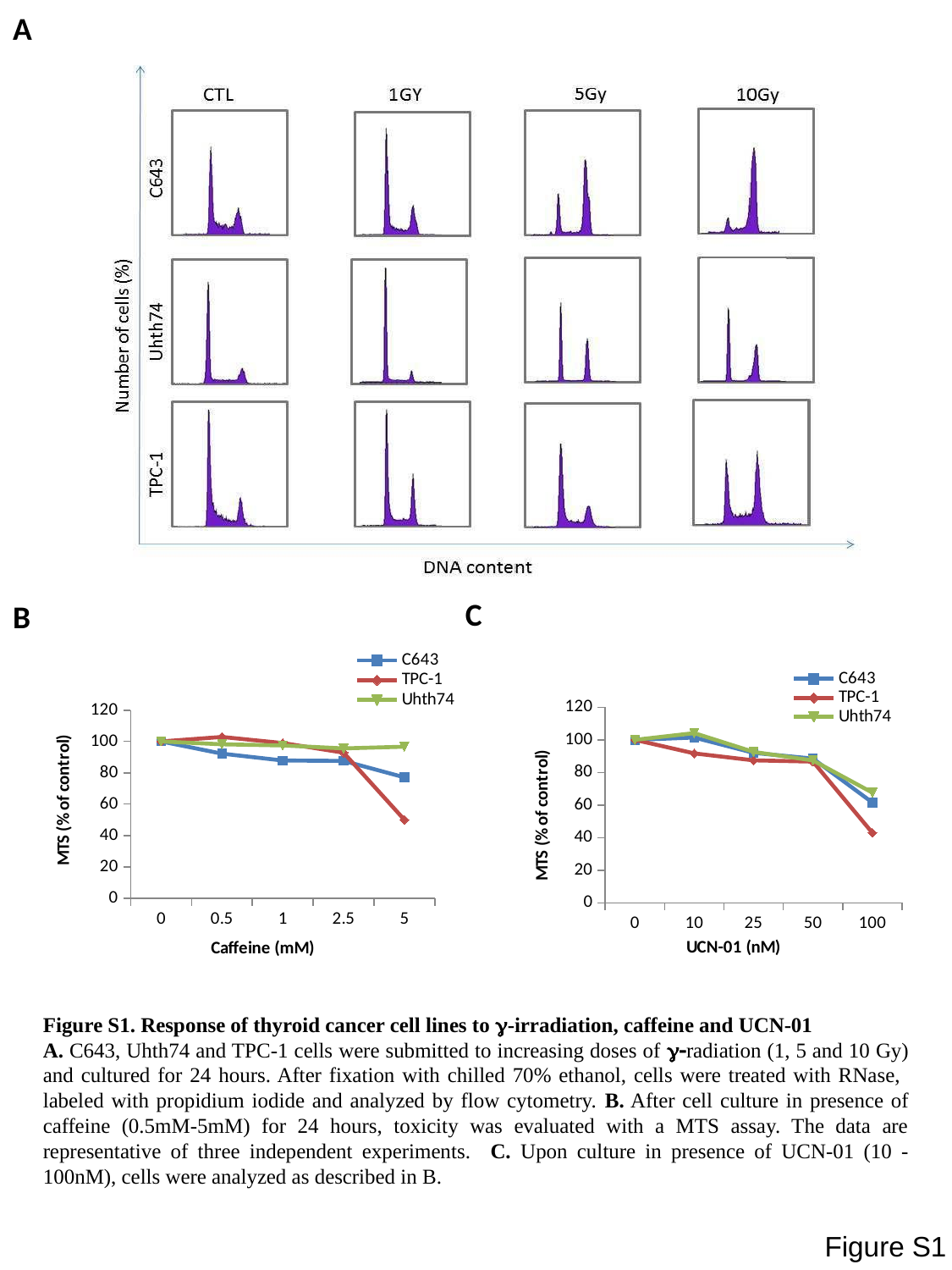

A
C
B
### Chart
| Category | C643 | TPC-1 | Uhth74 |
|---|---|---|---|
| 0 | 100.0 | 100.0 | 100.0 |
| 0.5 | 92.21099670165263 | 102.8765705825763 | 98.20709766676522 |
| 1 | 87.8577634405692 | 98.99144230731002 | 97.44567869258657 |
| 2.5 | 87.61244085334876 | 92.87499489512257 | 95.57395921048024 |
| 5 | 77.09105005125852 | 50.02578814530752 | 96.65520610643132 |
### Chart
| Category | C643 | TPC-1 | Uhth74 |
|---|---|---|---|
| 0 | 100.0 | 100.0 | 100.0 |
| 10 | 101.3983908837138 | 91.66858891801895 | 104.1208195877107 |
| 25 | 92.02137688589845 | 87.4185192826104 | 92.59166929858652 |
| 50 | 88.50020037360126 | 86.63819279622575 | 87.3603142309632 |
| 100 | 61.56912329927101 | 42.99239039784077 | 67.64072684346745 |Figure S1. Response of thyroid cancer cell lines to g-irradiation, caffeine and UCN-01
A. C643, Uhth74 and TPC-1 cells were submitted to increasing doses of g-radiation (1, 5 and 10 Gy) and cultured for 24 hours. After fixation with chilled 70% ethanol, cells were treated with RNase, labeled with propidium iodide and analyzed by flow cytometry. B. After cell culture in presence of caffeine (0.5mM-5mM) for 24 hours, toxicity was evaluated with a MTS assay. The data are representative of three independent experiments. C. Upon culture in presence of UCN-01 (10 -100nM), cells were analyzed as described in B.
Figure S1

## Slide 2
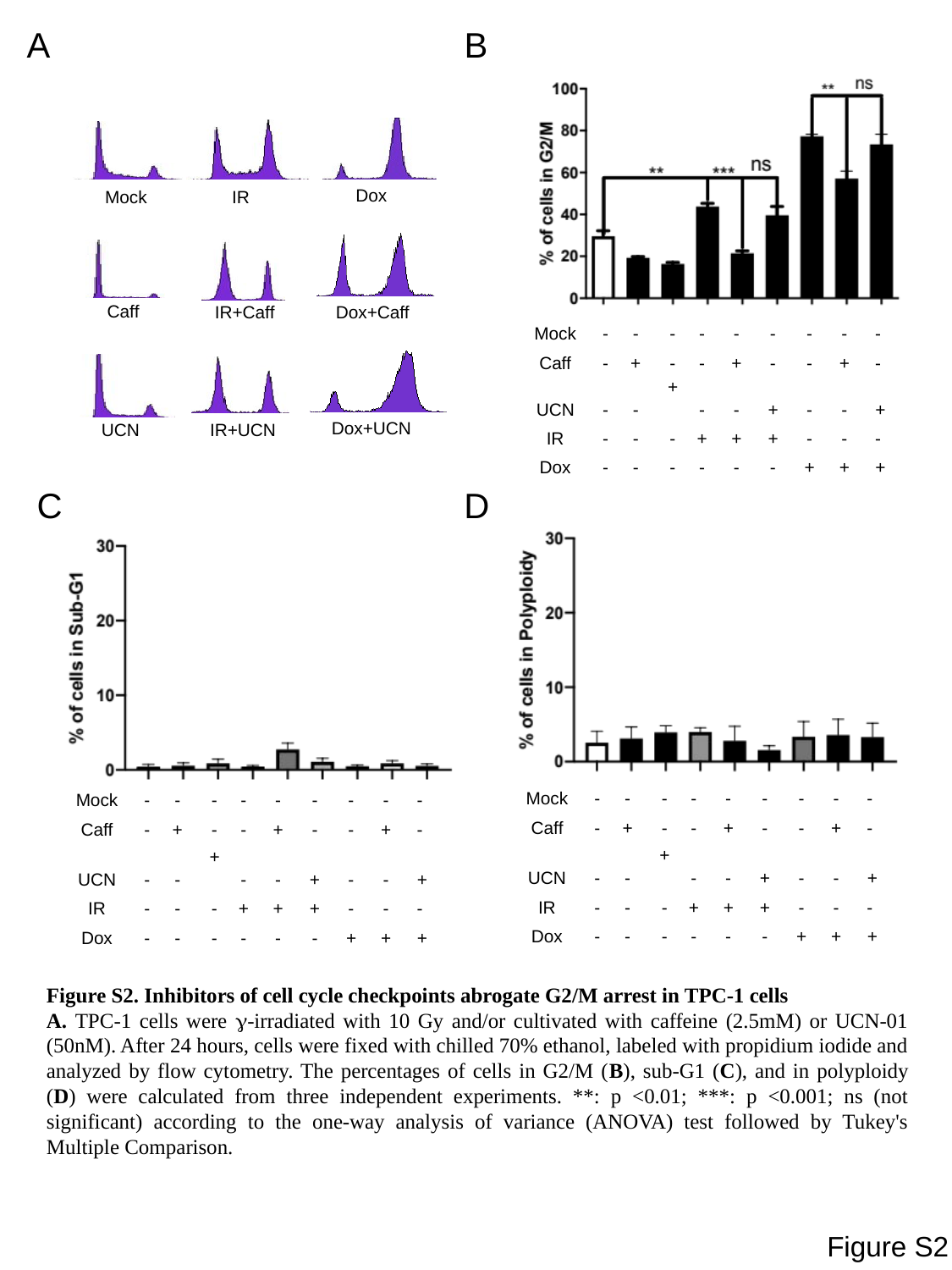

A
B
Dox
Mock
IR
Caff
IR+Caff
Dox+Caff
| Mock | - | - | - | - | - | - | - | - | - |
| --- | --- | --- | --- | --- | --- | --- | --- | --- | --- |
| Caff | - | + | - | - | + | - | - | + | - |
| UCN | - | - | + | - | - | + | - | - | + |
| IR | - | - | - | + | + | + | - | - | - |
| Dox | - | - | - | - | - | - | + | + | + |
Dox+UCN
UCN
IR+UCN
C
D
| Mock | - | - | - | - | - | - | - | - | - |
| --- | --- | --- | --- | --- | --- | --- | --- | --- | --- |
| Caff | - | + | - | - | + | - | - | + | - |
| UCN | - | - | + | - | - | + | - | - | + |
| IR | - | - | - | + | + | + | - | - | - |
| Dox | - | - | - | - | - | - | + | + | + |
| Mock | - | - | - | - | - | - | - | - | - |
| --- | --- | --- | --- | --- | --- | --- | --- | --- | --- |
| Caff | - | + | - | - | + | - | - | + | - |
| UCN | - | - | + | - | - | + | - | - | + |
| IR | - | - | - | + | + | + | - | - | - |
| Dox | - | - | - | - | - | - | + | + | + |
Figure S2. Inhibitors of cell cycle checkpoints abrogate G2/M arrest in TPC-1 cells
A. TPC-1 cells were g-irradiated with 10 Gy and/or cultivated with caffeine (2.5mM) or UCN-01 (50nM). After 24 hours, cells were fixed with chilled 70% ethanol, labeled with propidium iodide and analyzed by flow cytometry. The percentages of cells in G2/M (B), sub-G1 (C), and in polyploidy (D) were calculated from three independent experiments. **: p <0.01; ***: p <0.001; ns (not significant) according to the one-way analysis of variance (ANOVA) test followed by Tukey's Multiple Comparison.
Figure S2

## Slide 3
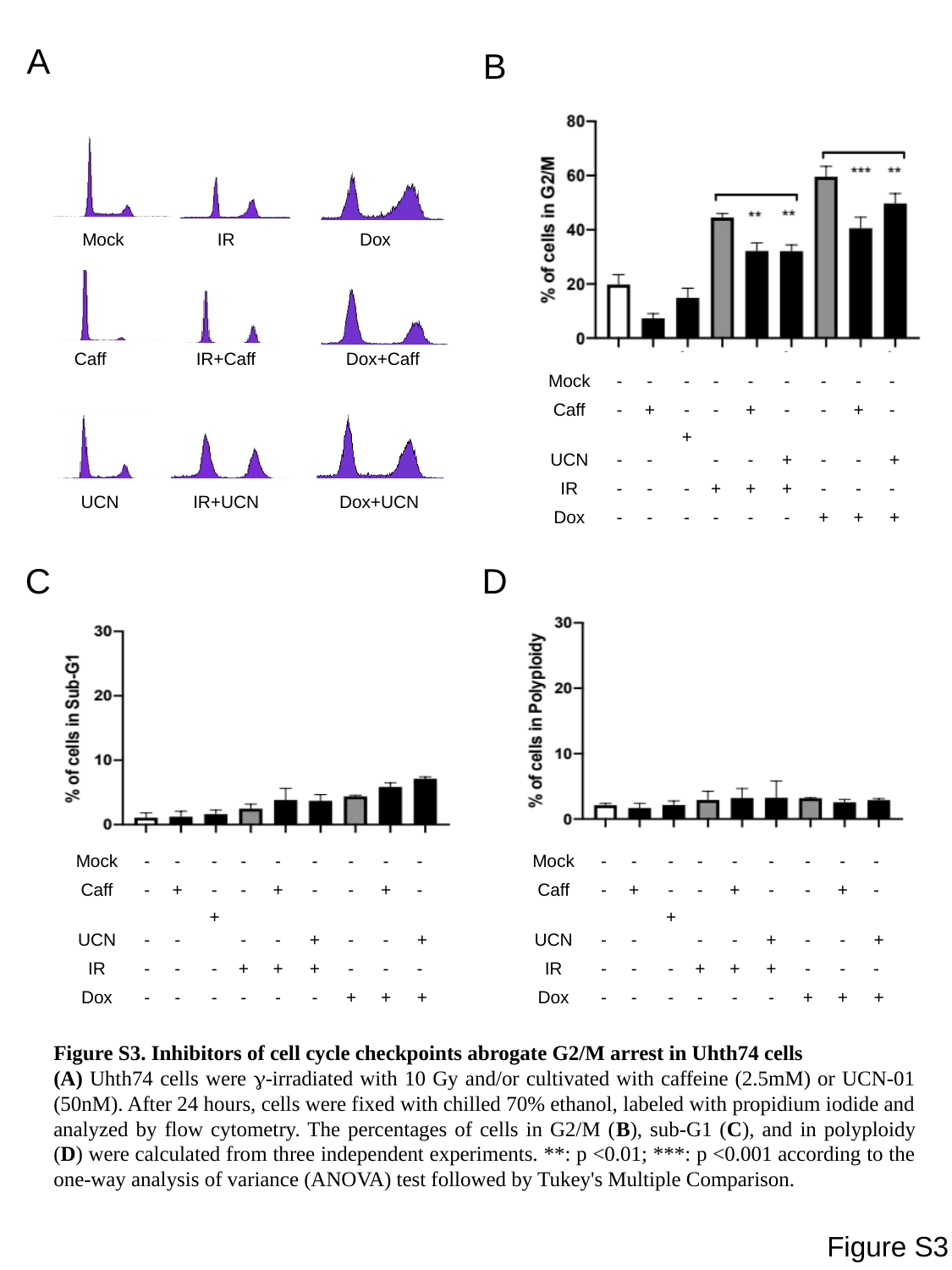

A
B
Mock
IR
Caff
IR+Caff
UCN
IR+UCN
Dox
Dox+Caff
Dox+UCN
| Mock | - | - | - | - | - | - | - | - | - |
| --- | --- | --- | --- | --- | --- | --- | --- | --- | --- |
| Caff | - | + | - | - | + | - | - | + | - |
| UCN | - | - | + | - | - | + | - | - | + |
| IR | - | - | - | + | + | + | - | - | - |
| Dox | - | - | - | - | - | - | + | + | + |
C
D
| Mock | - | - | - | - | - | - | - | - | - |
| --- | --- | --- | --- | --- | --- | --- | --- | --- | --- |
| Caff | - | + | - | - | + | - | - | + | - |
| UCN | - | - | + | - | - | + | - | - | + |
| IR | - | - | - | + | + | + | - | - | - |
| Dox | - | - | - | - | - | - | + | + | + |
| Mock | - | - | - | - | - | - | - | - | - |
| --- | --- | --- | --- | --- | --- | --- | --- | --- | --- |
| Caff | - | + | - | - | + | - | - | + | - |
| UCN | - | - | + | - | - | + | - | - | + |
| IR | - | - | - | + | + | + | - | - | - |
| Dox | - | - | - | - | - | - | + | + | + |
Figure S3. Inhibitors of cell cycle checkpoints abrogate G2/M arrest in Uhth74 cells
(A) Uhth74 cells were g-irradiated with 10 Gy and/or cultivated with caffeine (2.5mM) or UCN-01 (50nM). After 24 hours, cells were fixed with chilled 70% ethanol, labeled with propidium iodide and analyzed by flow cytometry. The percentages of cells in G2/M (B), sub-G1 (C), and in polyploidy (D) were calculated from three independent experiments. **: p <0.01; ***: p <0.001 according to the one-way analysis of variance (ANOVA) test followed by Tukey's Multiple Comparison.
Figure S3

## Slide 4
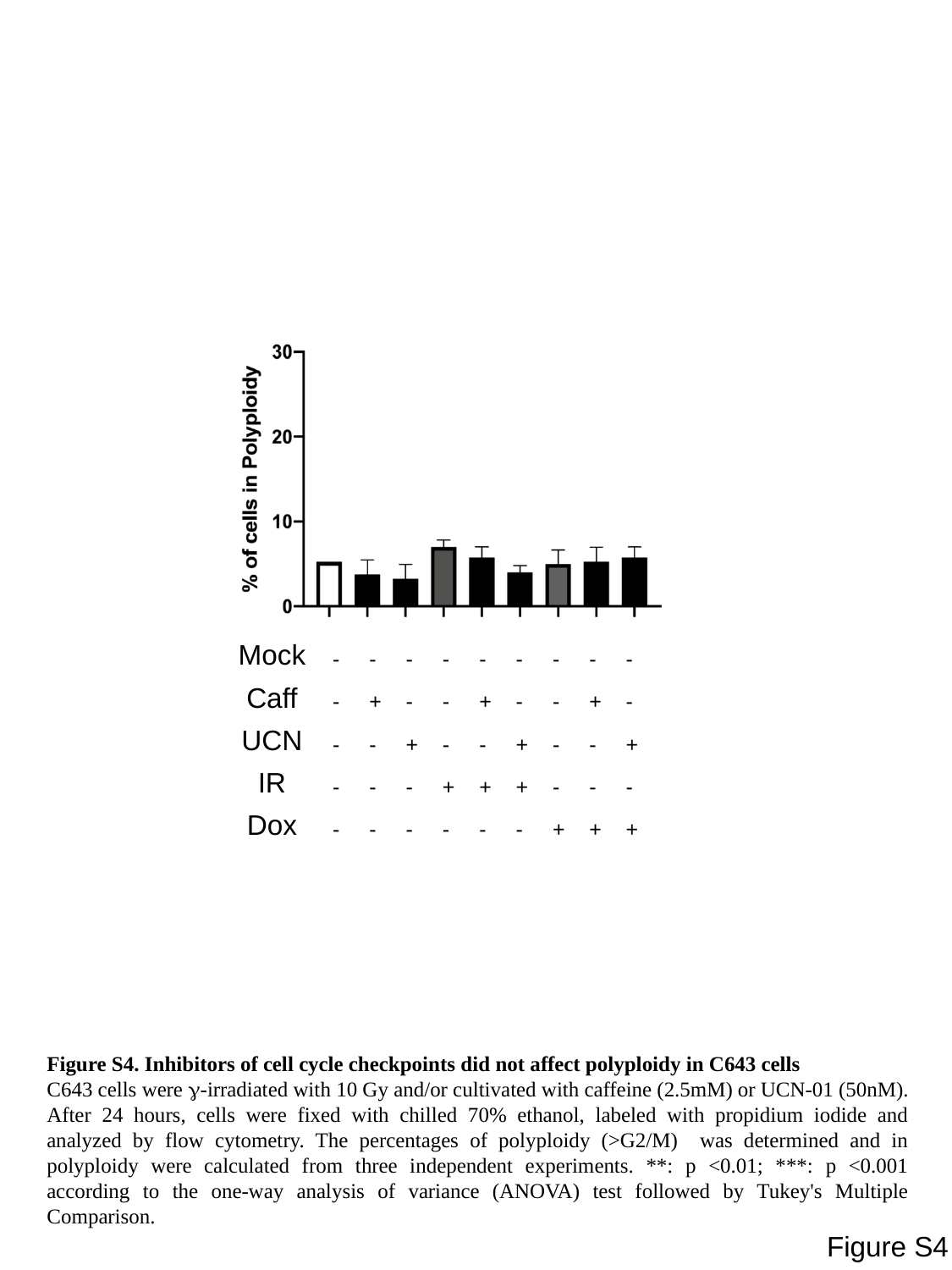

| Mock | - | - | - | - | - | - | - | - | - |
| --- | --- | --- | --- | --- | --- | --- | --- | --- | --- |
| Caff | - | + | - | - | + | - | - | + | - |
| UCN | - | - | + | - | - | + | - | - | + |
| IR | - | - | - | + | + | + | - | - | - |
| Dox | - | - | - | - | - | - | + | + | + |
Figure S4. Inhibitors of cell cycle checkpoints did not affect polyploidy in C643 cells
C643 cells were g-irradiated with 10 Gy and/or cultivated with caffeine (2.5mM) or UCN-01 (50nM). After 24 hours, cells were fixed with chilled 70% ethanol, labeled with propidium iodide and analyzed by flow cytometry. The percentages of polyploidy (>G2/M) was determined and in polyploidy were calculated from three independent experiments. **: p <0.01; ***: p <0.001 according to the one-way analysis of variance (ANOVA) test followed by Tukey's Multiple Comparison.
Figure S4

## Slide 5
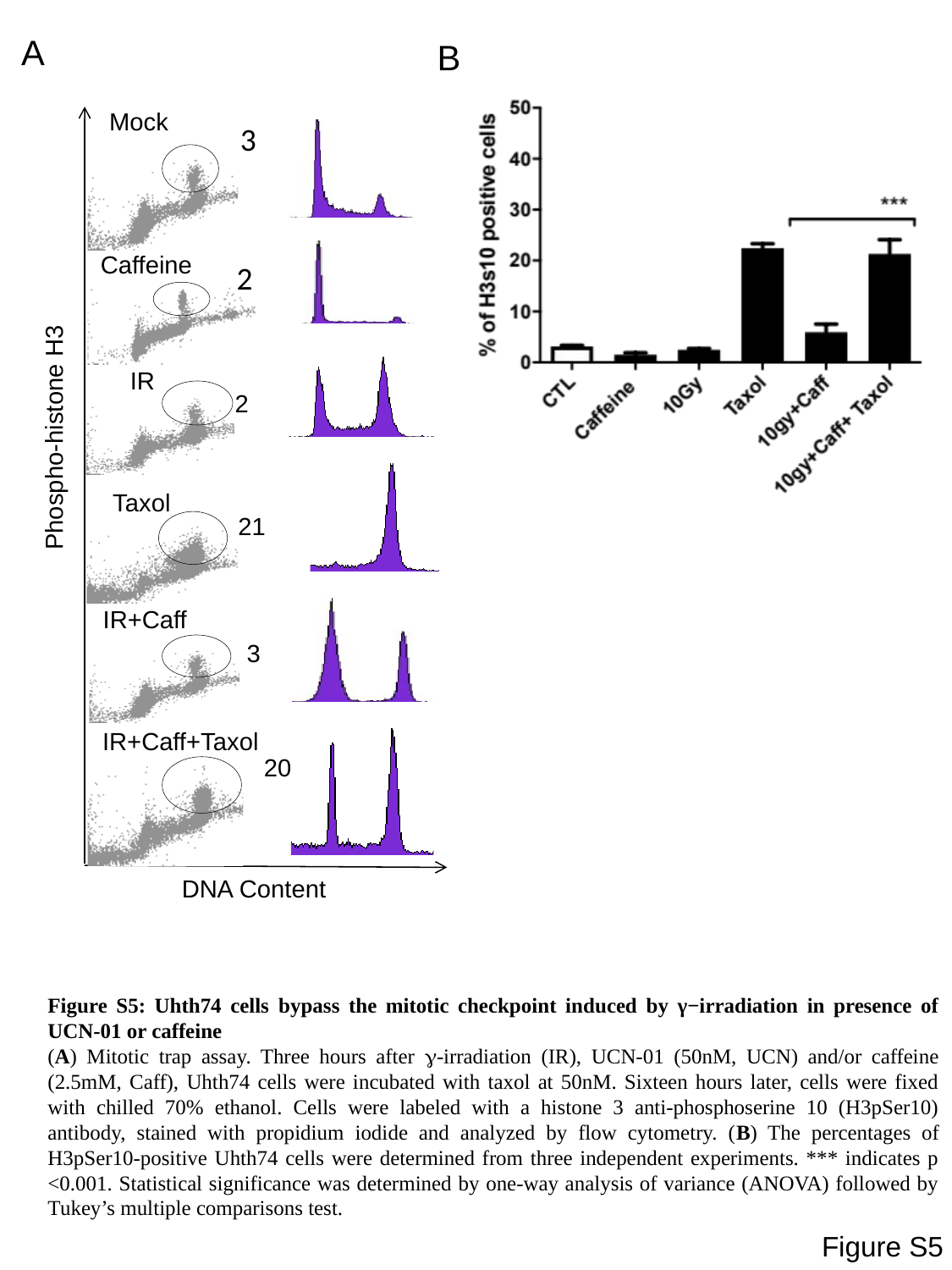

A
B
Mock
2
IR
2
21
3
20
3
Mock
Caffeine
2
Phospho-histone H3
Taxol
IR+Caff
IR+Caff+Taxol
DNA Content
Figure S5: Uhth74 cells bypass the mitotic checkpoint induced by γ−irradiation in presence of UCN-01 or caffeine
(A) Mitotic trap assay. Three hours after g-irradiation (IR), UCN-01 (50nM, UCN) and/or caffeine (2.5mM, Caff), Uhth74 cells were incubated with taxol at 50nM. Sixteen hours later, cells were fixed with chilled 70% ethanol. Cells were labeled with a histone 3 anti-phosphoserine 10 (H3pSer10) antibody, stained with propidium iodide and analyzed by flow cytometry. (B) The percentages of H3pSer10-positive Uhth74 cells were determined from three independent experiments. *** indicates p <0.001. Statistical significance was determined by one-way analysis of variance (ANOVA) followed by Tukey’s multiple comparisons test.
Figure S5

## Slide 6
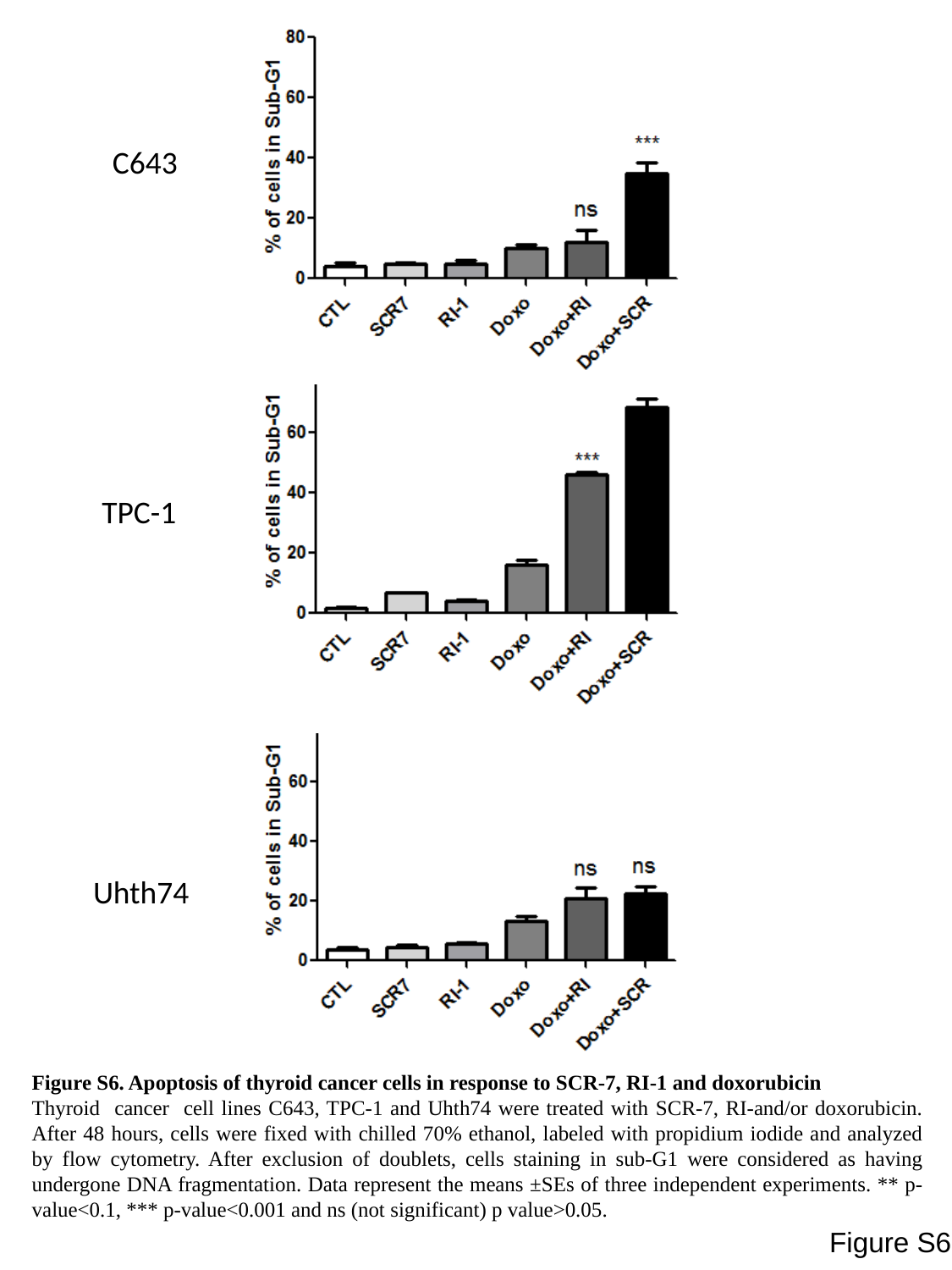

C643
TPC-1
Uhth74
Figure S6. Apoptosis of thyroid cancer cells in response to SCR-7, RI-1 and doxorubicin
Thyroid cancer cell lines C643, TPC-1 and Uhth74 were treated with SCR-7, RI-and/or doxorubicin. After 48 hours, cells were fixed with chilled 70% ethanol, labeled with propidium iodide and analyzed by flow cytometry. After exclusion of doublets, cells staining in sub-G1 were considered as having undergone DNA fragmentation. Data represent the means ±SEs of three independent experiments. ** p-value<0.1, *** p-value<0.001 and ns (not significant) p value>0.05.
Figure S6
